# Anemia and Iron Deficiency Anemia in Saudi Arabia: A Systematic Review of Prevalence and Contributing Factors

**DOI:** 10.3390/jcm15156074

**Published:** 2026-08-04

**Authors:** Farjah H. Algahtani, Hatoon Mohammed Ezzat, Mohammed K. Alabdulaali, Durria Ahmed Abdulmaged Ahmed

**Affiliations:** 1 Oncology Center, Epidemiology and Public Health Research Chair, Faculty of Medicine, King Saud University/King Saud Medical City, Riyadh 12373, Saudi Arabia; 2 Ministry of Health, Riyadh 12822, Saudi Arabia; hezzat@moh.gov.sa (H.M.E.); mal-abdul-aali@moh.gov.sa (M.K.A.); 3 Epidemiology and Public Health Research Chair, Faculty of Medicine, King Saud University, Riyadh 11321, Saudi Arabia; duahmed@ksu.edu.sa

**Keywords:** anemia, iron deficiency anemia (IDA), prevalence, hemoglobin, iron deficiency, Saudi Arabia

## Abstract

**Background:** Iron deficiency anemia (IDA) is among the most prevalent nutritional disorders worldwide and a growing public health concern among women in Saudi Arabia. Available Saudi evidence remains fragmented, with single-center studies that vary by population, region, and diagnostic criteria. This review evaluated the prevalence of anemia in Saudi Arabia, assessed gender-related differences across the lifespan, and highlighted local determinants and prevention strategies relevant to women. **Methods**: Eight cross-sectional studies (26,412 participants) reporting anemia prevalence in Saudi individuals aged ≥13 with both-sex data were identified across five databases (2007–2024), excluding chronic-disease and under-13 studies. Quality was appraised with the JBI checklist, and because of substantial heterogeneity, findings were synthesized narratively by population group (SWiM guideline) rather than pooled. **Results:** Anemia was consistently more prevalent in women than men across all population groups and regions. Female prevalence ranged from about 18–22% in older and community adults to over 65% in female university students, whereas male prevalence ranged from none detected to 28% in a hospital-based sample. The lowest mean hemoglobin (10.4 g/dL) occurred in young women. Between study heterogeneity was considerable (I^2^ > 99%) and persisted within sex subgroups, so findings were synthesized by population group rather than pooled into a single estimate. **Conclusions:** Anemia remains a significant public health challenge among women in Saudi Arabia, shaped by an interacting set of dietary, sociocultural, hereditary, and health-system determinants specific to the local context. Beyond confirming the female burden, this review consolidates fragmented national evidence, appraises its quality, and translates the findings into targeted recommendations: dietary counseling to address local inhibitors of iron absorption, screening during peak-risk periods, and integration with existing premarital screening and food-fortification infrastructure.

## 1. Introduction

Anemia is a prevalent and widespread health issue, especially among at-risk groups such as women, children, and individuals engaged in intense physical activity [1]. It commonly results from a mismatch between iron intake and the body’s physiological requirements, with young women particularly affected owing to menstrual iron loss [2]. Anemia is characterized by low blood hemoglobin (Hb) and has a significant negative effect on physical and mental performance, especially in children, expectant mothers, and newborns [3]. Pregnant women are at increased risk because of heightened iron needs, short inter-pregnancy intervals, and inadequate dietary intake, which can lead to complications such as intrauterine growth restriction [4] and low birth weight [5].

Iron deficiency progresses through three stages: iron depletion, indicated by reduced serum ferritin; iron deficiency without anemia, characterized by lower serum iron, ferritin, and transferrin saturation, with increased iron-binding capacity; and iron-deficiency anemia (IDA), in which reduced hemoglobin impairs oxygen delivery and tissue function [6]. IDA is the most common nutritional deficiency globally [7], and women are at greater risk because of increased iron demand during development, pregnancy, and reproductive life, compounded by menstrual iron loss [8]. In 2021, anemia affected 24.3% of the global population, with a higher incidence in females (31%) than in males (17.5%) [9]. The World Health Organization (WHO) defines iron deficiency in healthy adults as serum ferritin below 15 μg/L, rising to below 70 μg/L in the presence of infection or inflammation [10].

A global study across 204 countries reported anemia prevalence of 22.8% in 2019, down from 27.0% in 1990, although the absolute number of cases rose with population growth. By WHO severity thresholds (mild, moderate, and severe, defined by hemoglobin bands), most cases were mild to moderate [11]. Women of reproductive age remain a primary concern under Global Nutrition Target 2. A prior systematic review in Saudi Arabia found that anemia was more prevalent in females, with contributing factors including menstruation, pregnancy, and dietary practices [12].

**Figure 1:**
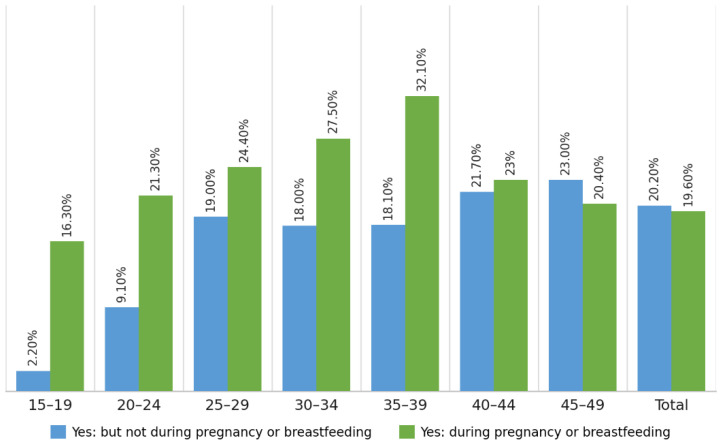
Percentage of anemia among women in Saudi Arabia by age group, during pregnancy or breastfeeding versus outside these conditions (data source: Statista, 2023 [13]; secondary compilation).

Figure 1 shows the reported prevalence of anemia among women in Saudi Arabia in 2023, categorized by age group and maternal condition. Across age groups, prevalence during pregnancy or breastfeeding generally increased with maternal age, and a substantial burden was also evident among women outside these conditions in the older reproductive-age bands. The figure illustrates that anemia in Saudi women is not confined to pregnancy but extends across the reproductive lifespan [13].

### 1.1. Multifactorial Causes and Contributing Conditions of Anemia

The WHO defines anemia in individuals aged ≥15 years as hemoglobin below 13.0 g/dL for men and below 12.0 g/dL for women [14]. Diagnostic thresholds vary with age, sex, and physiological state, including pregnancy, lactation, rapid growth, and intense physical activity [15,16,17]. Anemia can arise from impaired nutrient absorption (e.g., coeliac disease [18], *Helicobacter pylori* infection [19], parasitic infestation), nutritional deficiencies, and hereditary red-cell disorders [20]. Iron deficiency, usually due to inadequate dietary intake, is the leading nutritional cause, while deficiencies in vitamin A, folate, vitamin B12, and other micronutrients also impair hemoglobin synthesis [21]. Sociodemographic factors—age, sex, education, and financial status—and genetic predispositions contribute, with menstrual blood loss a significant factor in young women [22]. Poor dietary habits, particularly irregular breakfast consumption, low intake of iron-rich foods, and limited nutritional awareness, have been identified as contributors among Saudi female students [23].

This review focuses on hemoglobin-defined anemia and, where the primary studies report it, specifically on iron-deficiency anemia. Several included studies reported overall anemia without distinguishing IDA; this distinction is retained throughout and addressed in Section 5.

### 1.2. Anemia in Saudi Arabia: Current Evidence and Rationale

Anemia, particularly iron deficiency, is among the most common nutritional conditions in Saudi Arabia, yet reported prevalence varies widely across studies—from low single digits in some male groups to over 70% in female students and pregnant women (Table 1). A prior systematic review estimated IDA prevalence between 9.8% and 75.3% across Saudi population groups [12]. Much of this variation reflects genuine differences in the populations sampled and in the diagnostic criteria applied rather than a single underlying national rate.

Across almost every population studied, prevalence is markedly higher in women than in men. Among university and health-science students, female prevalence ranged from roughly 13% to over 65%, compared with low single- to low double-digit figures in males [22,24,25,26,27,28], with a comparable female excess in adolescents [29] and community adults [30]. This disparity is consistently attributed to the combination of menstrual and reproductive iron loss with dietary inadequacy: menorrhagia and prolonged cycles [31], high parity and short birth intervals among pregnant women [32], skipped breakfasts, low intake of iron-rich foods, and tea or coffee consumed with meals [28,33], and low total iron intake—Al-Assaf reported intakes below recommended levels in around 95% of women versus a small minority of men [30].

The dominant drivers shift across the life course. In adolescents and young women, nutritional and menstrual factors predominate [26,29,33]; in pregnancy, prevalence is high and clinically consequential, with maternal anemia linked to low birth weight and growth restriction [32]. In older adults, the picture differs, with prevalence tied more to chronic disease, reduced marrow activity, impaired nutrient absorption, and limited healthcare access among lower-income individuals than to reproductive loss [34]. Inherited contributors are also regionally relevant, with hemoglobinopathy traits detected through national student screening [35]. Studies in young children that showed a male-predominant microcytic pattern [36] fall outside the scope of the present review.

Critically, these studies are not directly comparable. They use varying hemoglobin thresholds and, when reported, different serum ferritin cutoffs; some report iron deficiency, others IDA, and others hemoglobin-defined anemia alone; and their sampling frames span community surveys, university convenience samples, and hospital attendees. This methodological and population heterogeneity, rather than any single true figure, accounts for much of the wide range in prevalence and motivates the structured, quality-appraised synthesis undertaken in this review.

**Table 1:** Summary of anemia prevalence and key findings from Saudi studies cited as background.

References	Study Objective	Anemia Prevalence	Key Findings
Hamali et al. [24]	Anemia among Jazan University students	4.7% M, 67.35% F	High female prevalence from low dietary iron; 26.54% of anemic females had low ferritin (IDA).
Abdelrahem et al. [33]	Hemoglobin and dietary habits in nursing students	59% F	Irregular eating and inadequate intake of iron-rich foods were significant contributors.
Hakami et al. [25]	Nutritional anemia among Jazan University students	8.8% M, 21% F (low ferritin)	88% of females had low serum iron; folate/B12 deficiency was uncommon.
Owaidah et al. [35]	National survey of IDA among Saudi students	ID 28.6%, IDA 10.7%	Cases are predominantly female (94.1% of IDA); dietary intake and hemoglobinopathy traits are implicated.
AlQuaiz et al. [31]	Risk factors for IDA in women of childbearing age	30–56% F	Menorrhagia and low meat/vitamin C intake were significant contributors.
Alhamed et al. [12]	Systematic review of IDA in Saudi Arabia	9.8–75.3%	High female burden from menorrhagia and low dietary iron.
Essawi et al. [26]	Anemia and academic achievement	52.5% F	Positive association between hemoglobin and academic performance.
Almasmoum et al. [27]	IDA among female medical students	13% F	Low iron intake is common; most cases are microcytic.
Al-Sayes et al. [28]	Iron deficiency and IDA among female students	23.9% F	Associated with poor dietary habits and low meat intake.
AlSheikh et al. [22]	Anemia among female medical students	38.3% F	No significant correlation with dietary or gynecological factors.
Alharbi et al. [36]	Anemia in young children, Albaha	81.4% M, 14.1% F (of cases)	Microcytic hypochromic anemia; excluded from the present review (children).
Salma U. et al. [32]	Anemia in pregnant women, Aljouf	77.9% moderate	Linked to low birth weight and growth restriction, short birth intervals, and multiparity.
Alsaeed et al. [34]	Anemia in elderly individuals	12.9% overall	Linked to chronic disease, reduced marrow activity, and nutrient absorption.
Al-Assaf et al. [30]	Iron intake and gender-based anemia	none M, 21.6% F	Economic disparities and limited access to iron-rich foods drove the female burden.

## 2. Materials and Methods

### 2.1. Inclusion and Exclusion Criteria

This systematic review included quantitative studies reporting the prevalence of anemia in Saudi Arabia among participants aged ≥13 years. Studies were required to report data for both males and females within the same study to allow comparison of prevalence between sexes. Studies of anemia secondary to comorbidities—heart failure, chronic kidney disease, cancer, or other chronic illness—were excluded, as were studies restricted to infants and children under 13 years. Only studies published in English were included. Studies that did not meet the both-sex requirement (including several female-only cohorts) are cited in the Introduction as background context but were not part of the quantitative synthesis; the eight studies included in the synthesis all reported both sexes.

### 2.2. Search Strategy

**Figure 2:**
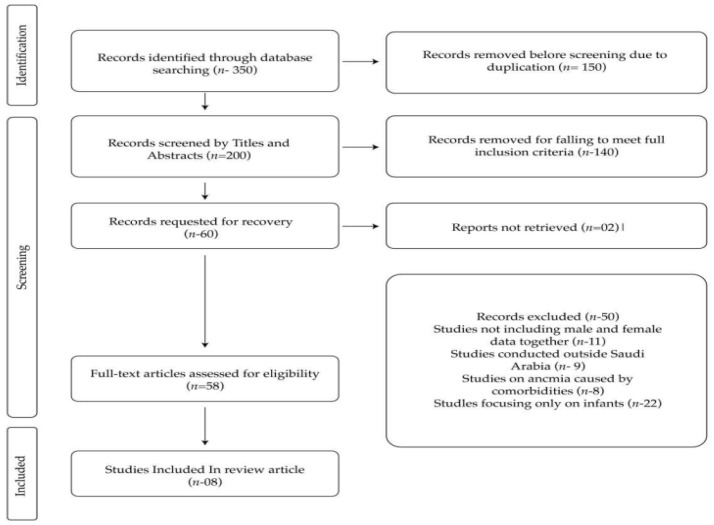
PRISMA Flow Diagram.

Studies published between 2007 and 2024 were searched across PubMed (U.S. National Library of Medicine, Bethesda, MD, USA), Scopus (Elsevier B.V., Amsterdam, The Netherlands), Google Scholar (Google LLC, Mountain View, CA, USA), the Saudi Digital Library (Riyadh, Saudi Arabia), and Web of Science (Clarivate Plc, London, United Kingdom). The search used the terms "anemia," "iron deficiency anemia," "prevalence," "hemoglobin," and "iron deficiency." It covered the entire period continuously; the lack of studies from 2012–2014 reflects the search results, not a specific exclusion. The PRISMA flow diagram for selecting studies is shown in Figure 2.

### 2.3. Quality Appraisal

The methodological quality of the included studies was independently appraised using the Joanna Briggs Institute (JBI) Critical Appraisal Checklist for Studies Reporting Prevalence Data. This tool was selected over alternatives (Newcastle–Ottawa Scale, AXIS, Cochrane RoB 2, ROBINS-I) because it is purpose-designed for prevalence studies and evaluates the sampling frame, recruitment, sample size, condition measurement, and statistical analysis. Each study was rated across the nine checklist items and assigned an overall risk-of-bias judgment.

### 2.4. Data Synthesis

Given substantial heterogeneity across the included studies in participant characteristics, age distribution, study settings, and diagnostic criteria, quantitative pooling of prevalence and hemoglobin values was considered inappropriate. Between-study heterogeneity in prevalence was quantified using Cochran’s Q and the I^2^ statistic on Freeman–Tukey double-arcsine-transformed proportions. Given the extent of heterogeneity observed, estimates were synthesized narratively by population group in accordance with the Synthesis Without Meta-analysis (SWiM) guideline rather than pooled. Findings are presented by population group and, within each, by sex.

## 3. Results

### 3.1. Study Selection

The search and selection followed PRISMA guidance (Figure 2); the completed PRISMA 2020 checklist is available as Supplementary Material (Table S1). Of 350 records identified, 150 duplicates were removed, and 200 were screened by title and abstract, of which 140 were excluded. Sixty full texts were sought; two could not be retrieved, and 58 were assessed for eligibility. Fifty were excluded—for not reporting combined male and female data (*n* = 11), being conducted outside Saudi Arabia (*n* = 9), reporting anemia secondary to comorbidities (*n* = 8), or focusing on infants and young children (*n* = 22) leaving eight studies for synthesis.

### 3.2. Characteristics of Included Studies

The eight included studies were cross-sectional and spanned 2007 to 2024, together comprising 26,412 participants (11,766 males [44.5%] and 14,646 females [55.5%]). Their designs, settings, and diagnostic criteria are summarized in Table 2. Populations ranged from adolescents aged 13 years to adults aged 89 years and were drawn from the Central (Riyadh), Western (Makkah), Southern (Jazan), and multiple pooled regions. Notably, a single hospital-based study (Arbaeen, 2022 [37]) contributed 21,464 participants—roughly 81% of the combined sample. As a result, any aggregate figure would be dominated by a single clinical population, reinforcing the need for a stratified rather than a pooled synthesis. The studies also differed in case definition, applying varying hemoglobin thresholds and, where measured, different serum ferritin cutoffs, with some reporting iron deficiency or iron-deficiency anemia rather than hemoglobin-defined anemia.

### 3.3. Methodological Quality

The methodological quality of the eight studies was appraised using the JBI Critical Appraisal Checklist for Prevalence Studies (Table 3). Two studies were rated as low risk of bias (Alquaiz 2015 [29]; Owaidah 2020 [35]), reflecting probability-based sampling frames designed to represent their target populations. Five were rated as moderate (Alsaeed 2011 [34]; Arbaeen 2022 [37]; Hamali 2020 [24]; Hakami 2024 [25]; Shaheen 2022 [38]), and one was rated as high risk of bias (Al-Assaf 2007 [30]) owing to a small convenience sample with limited description of recruitment. The principal concerns across studies were non-random or convenience sampling frames, single-institution recruitment that limited representativeness, and inconsistent case definitions. Two studies (Arbaeen [37]; Shaheen [38]) drew on clinical populations whose sampling frames do not represent the general population, and their estimates are therefore interpreted accordingly.

### 3.4. Prevalence of Anemia by Population Group

**Figure 3:**
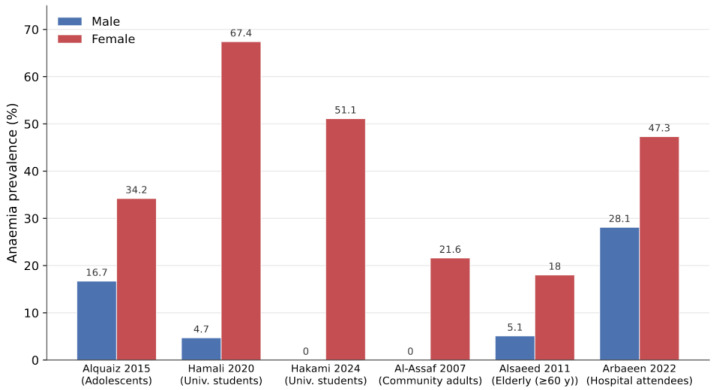
Anemia prevalence by sex [24,25,29,30,34,37].

To avoid overgeneralization across non-comparable populations, prevalence is synthesized by population group and, within each, by sex (Figure 3). Formal testing confirmed considerable between-study heterogeneity in prevalence (Cochran’s Q = 675, df = 5, *p* < 0.001; I^2^ > 99%), which persisted within sex subgroups (I^2^ = 93% in women, 98% in men). These values, far above the conventional 75% threshold for considerable heterogeneity, indicate that a single pooled estimate would be uninterpretable and support the stratified synthesis presented below. A consistent female excess was evident across all groups.

**Adolescents.** In the only community-based adolescent study (Alquaiz, 2015 [29]; Riyadh; *n* = 495), anemia affected 34.2% of girls and 16.7% of boys aged 13–18 years. Higher risk was associated with menstrual iron loss, low dietary iron, obesity, low socioeconomic status and maternal education, and a family history of iron-deficiency anemia, which raised risk three- to six-fold.

**University students.** Three studies sampled university students and found the steepest sex disparity. In Jazan, Hamali (2020 [24]; *n* = 134) reported anemia in 67.4% of women versus 4.7% of men, and Hakami (2024 [25]; *n* = 158) reported that 51.1% of women were anemic (mean hemoglobin 10.4 g/dL), with no anemia detected in men. The national study by Owaidah (2020 [35]; *n* = 981, four regions) reported iron deficiency in 28.6% and iron-deficiency anemia in 10.7% of students, with cases overwhelmingly female (94.1% of IDA cases). Reported drivers were behavioral and dietary: skipping breakfast, irregular meals, and low intake of iron-rich foods, alongside detectable hemoglobinopathy traits (β-thalassemia and sickle-cell trait).

Community adults. Among healthy Riyadh adults (Al-Assaf, 2007 [30]; *n* = 234), anemia was present in 21.6% of women, while none was detected in men. Inadequate dietary iron intake was near-universal among women (approximately 95% below recommended levels), and women from lower-income households had reduced access to iron-rich foods.

**Hospital-based populations.** The large Makkah outpatient study (Arbaeen, 2022 [37]; *n* = 21,464) reported an overall prevalence of 38.7%–47.3% in women and 28.1% in men. As a care-seeking clinical sample, these figures are higher than community estimates and should not be interpreted as population prevalence.

**Older adults.** Among community-dwelling elderly aged ≥60 years in Riyadh (Alsaeed, 2011 [34]; *n* = 1558), overall prevalence was 12.9%, higher in women (18%) than men (5.1%). In this group, the drivers differed, reflecting chronic disease, reduced marrow activity, impaired nutrient absorption, and limited healthcare access among lower-income individuals rather than reproductive iron loss.

Multi-region reference cohort. In a tertiary hospital reference-interval cohort spanning three regions (Shaheen, 2022 [38]; *n* = 1388; 85% Central), anemia was incidental, with an overall prevalence of 10.6%, markedly higher among women (22.5%) than among men.

Figure 3. Prevalence of anemia by sex across the included Saudi studies, ordered by age. Values are hemoglobin-defined prevalence as reported in each source (Al-Assaf 2007 [30] and Hakami 2024 [25] detected no anemia in males). Owaidah (2020) [35] and Shaheen (2022) [38] are presented in Table 2 but omitted here because the former reports iron-deficiency cases rather than hemoglobin-defined prevalence, and the latter is a reference-interval cohort, neither of which is directly comparable on this axis.

### 3.5. Cross-Study Patterns

Three patterns held across the synthesis. First, women consistently bore a higher burden than men in every population and region, with the greatest disparity among reproductive-age students, reflecting the compounding of menstrual and reproductive iron demands with dietary inadequacy. Second, absolute prevalence tracked the sampling frame as much as geography, with the clinical Makkah sample far exceeding community and reference cohorts. Third, the studies were not directly comparable: Owaidah reports iron-deficiency cases rather than hemoglobin-defined prevalence, and Shaheen is a reference cohort in which anemia was incidental, so neither is combined with the hemoglobin-defined estimates. These observations, together with documented variation in diagnostic thresholds and extreme heterogeneity statistics, indicate that the wide prevalence range reflects methodological and population heterogeneity rather than a single national rate.

## 4. Discussion

Anemia is a major global health issue, particularly in low-resource settings, where it contributes to adverse health outcomes and disproportionately affects women. The WHO defines anemia by hemoglobin thresholds that vary by sex and physiological state [14]. Although anemia has many causes, iron deficiency is the most common; comorbid chronic conditions, limited access to healthcare, low education, financial hardship, poor diet, closely spaced pregnancies, and insufficient micronutrient supplementation all increase risk.

Across the included studies, anemia was consistently more prevalent among women than men in every population group, with correspondingly lower mean hemoglobin among women and the lowest values in young women. This female excess is consistent with international evidence, including a study in Ethiopia involving 970 women aged 15–49, which reported that 29.4% had anemia, 32.1% had iron deficiency, and 18.0% had iron-deficiency anemia, with the highest prevalence among women aged 31–49 years [24].

A study by Hwang et al. [39] in the United States, involving 87,554 participants, found that women had a significantly higher prevalence of anemia than men (PR = 1.98, 95% CI 1.83–2.13) and that sex modified the relationship between anemia and race [39]. Similarly, a study of 2520 women of reproductive age, 1044 men, and 1528 children reported prevalence rates of 17% in women, 8% in men, and 22% in children. Folate and vitamin B12 deficiencies were common, underscoring the value of folic acid fortification and targeted iron supplementation [40]. AlFaris et al. [41] reported anemia in 40% of non-pregnant women in Riyadh, attributing the burden to low dietary iron intake, poor socioeconomic status, and inadequate nutrient intake.

In the present synthesis, the prevalence of anemia varied across the life course. Among adults aged 18–65 in Riyadh, Al-Assaf [30] recorded no anemia in adult males and 21.6% in females, while among those over 60 years (Alsaeed et al. [34]), prevalence was higher in females (18%) than males (5.06%), reflecting aging-related health issues and chronic disease. In hospital outpatients aged 15–89 years (Arbaeen et al. [37]), prevalence was substantial in both sexes (28.1% in males and 47.1% in females in Makkah), consistent with a care-seeking clinical population (Table 2). These life-course patterns echo international findings that anemia prevalence rises with age and frailty [42,43,44].

### Local and Contextual Determinants of Anemia in Saudi Arabia

**The elevated anemia burden documented in this review, particularly among women, is shaped by determinants specific to the Saudi environment, culture, and health behaviors rather than by physiology alone.**

**Environmental and structural factors.** Two features of the Saudi setting stand out. First, hereditary hemoglobinopathies contribute an inherited component distinct from nutritional deficiency: Owaidah et al. [35] detected β-thalassemia trait and sickle-cell trait in approximately 1.3% and 7% of participants, respectively, and Hamali et al. [24] noted indices suggestive of thalassemia carriage among male students. These conditions are the focus of the national premarital screening program, which provides an existing platform for detection. Second, the national folate food-fortification policy, noted by Hakami et al. [25], illustrates how population-level measures modify micronutrient status. Prevalence also varied by region across the included studies [35,38], reflecting differences in diet and healthcare access.

**Sociocultural factors.** Reproductive patterns are a central driver of the female excess: menstrual iron loss, high parity, and pregnancy-related demand were repeatedly implicated [29,35,37], with Owaidah et al. [35] quantifying menstrual losses of 15–30 mg of iron per cycle. Socioeconomic position compounded this risk, with Alquaiz et al. [29] reporting higher risk among adolescents from low-income households and those born to less-educated mothers, and a threefold-to-sixfold increase in risk with a family history of iron-deficiency anemia. The same study documented a nutrition transition in which the traditional Saudi diet of dates, milk, rice, vegetables, and fish has been increasingly displaced by energy-dense, iron-poor foods.

**Behavioral factors.** Modifiable dietary behaviors recurred across the student and adult cohorts: breakfast skipping and irregular meals [24,29], low intake of red meat and other iron-rich foods, and inadequate total dietary iron—Al-Assaf [30] found intake below recommended levels in 95.1% of women. Cigarette smoking [30] and obesity with physical inactivity [29] were additional reported correlates. Taken together, these environmental, sociocultural, and behavioral layers indicate that the high female burden is multifactorial and that effective intervention will need to combine dietary counseling, reproductive health measures, and the existing fortification and screening infrastructure rather than relying on supplementation alone.

## 5. Limitations

Several limitations should be acknowledged. First, the included studies were highly diverse across clinical and methodological dimensions, including population characteristics, sampling methods, and diagnostic thresholds, resulting in substantial statistical heterogeneity (I^2^ > 99%) that precluded quantitative synthesis. Consequently, the findings are descriptive and do not provide a single national estimate. Second, most demographic subgroups were represented by only one study, so conclusions about within-group differences are based on limited evidence and should be interpreted cautiously. Third, many primary studies reported overall hemoglobin-defined anemia without distinguishing iron-deficiency anemia from other causes, thereby limiting the specificity of conclusions for IDA. Fourth, none of the studies provided prevalence data by occupation or socioeconomic status, making formal subgroup analysis impossible; socioeconomic insights could only be summarized narratively. Fifth, some student studies relied on convenience samples and included populations with high consumption of caffeine and energy drinks, known confounders of dietary and health status [45,46,47], potentially limiting the generalizability of the findings. Lastly, because data were collected between 2007 and 2024, recent socioeconomic and dietary changes may not be fully reflected.

## 6. Conclusions

Anemia is a major public health concern among women in Saudi Arabia, shaped by an interacting set of biological, dietary, sociocultural, and structural determinants. Women of reproductive age and adolescents are particularly vulnerable owing to heightened physiological demand and nutritional inadequacy. Beyond confirming this burden, the present review consolidates fragmented national evidence, appraises its methodological quality, and makes the underlying heterogeneity explicit rather than averaging it into a single figure.

These determinants translate into three concrete priorities. First, dietary and behavioral counseling should target the specific patterns identified here—skipping breakfast, low intake of iron-rich foods, and drinking tea or coffee with meals—with particular focus on adolescent girls and women of reproductive age. Second, anemia and iron-status assessments should be integrated into the existing national premarital screening and folate fortification infrastructure, enabling capture of both nutritional and hereditary (thalassemia and sickle cell) contributors through platforms already in place. Third, future primary studies should report prevalence disaggregated by region and socioeconomic status, so that the environmental and structural disparities suggested by the current evidence can be quantified and addressed.

## Figures and Tables

**Table 2:** Characteristics of the eight included studies.

Study (Year)	Region	Population	N (M/F)	Case Definition	Prevalence (M/F/Overall)
Alquaiz 2015 [29]	Central (Riyadh)	Community adolescents, 13–18 y	495 (203/292)	Hb < 12 g/dL	16.7%/34.2%/27.1%
Al-Assaf 2007 [30]	Central (Riyadh)	Healthy adults	234 (132/102)	Hb/Hct (M < 13, F < 12)	none/21.6%/—
Alsaeed 2011 [34]	Central (Riyadh)	Elderly, ≥60 y	1558	WHO Hb thresholds	5.1%/18.0%/12.9%
Arbaeen 2022 [37]	Western (Makkah)	Hospital outpatients, 15–89 y	21,464 (9444/12,020)	WHO Hb thresholds	28.1%/47.3%/38.7%
Hamali 2020 [24]	Southern (Jazan)	University students,18–25 y	134 (85/49)	Hb (M < 13, F < 12)	4.7%/67.4%/—
Hakami 2024 [25]	Southern (Jazan)	University students18–25 y	158 (68/90)	Hb-defined	0%/51.1%/29.1%
Owaidah 2020 [35]	National (4 regions)	University students, >18 y	981 (474/507)	Hb + ferritin (ID/IDA)	ID 28.6%; IDA 10.7% (94.1% of IDA female)
Shaheen 2022 [38]	3 regions (85% Central)	Tertiary-hospital reference cohort	1388 (747/641)	Hb-defined (incidental)	—/22.5%/10.6%

**Table 3:** JBI Critical Appraisal Checklist for Studies Reporting Prevalence Data—item-level judgments for the eight included studies.

Study	Q1	Q2	Q3	Q4	Q5	Q6	Q7	Q8	Q9	Overall
Alquaiz 2015 [29]	Y	Y	Y	Y	Y	Y	Y	Y	Y	Low
Owaidah 2020 [35]	Y	Y	Y	Y	Y	Y	Y	Y	Y	Low
Alsaeed 2011 [34]	U	U	Y	U	Y	Y	Y	U	U	Moderate
Arbaeen 2022 [37]	N	U	Y	Y	Y	Y	Y	Y	Y	Moderate
Hamali 2020 [24]	U	N	N	Y	Y	Y	Y	Y	U	Moderate
Hakami 2024 [25]	U	N	N	Y	Y	Y	Y	Y	U	Moderate
Shaheen 2022 [38]	N	U	Y	Y	Y	Y	Y	Y	U	Moderate
Al-Assaf 2007 [30]	U	N	N	Y	Y	Y	Y	U	U	High

Y = yes; N = no; U = unclear. Q1, sampling frame appropriate; Q2, participants sampled appropriately; Q3, adequate sample size; Q4, subjects and setting described in detail; Q5, data analysis covered the identified sample; Q6, valid methods used to identify the condition; Q7, condition measured in a standard, reliable way; Q8, appropriate statistical analysis; Q9, adequate response rate.

## Data Availability

The data supporting this review are available from the corresponding author upon reasonable request.

## Supplementary Materials

The following supporting information can be downloaded at: https://www.mdpi.com/article/10.3390/jcm15156074/s1, Table S1: Completed PRISMA 2020 Checklist. Reference [48] is cited in Supplementary Materials.

## Abbreviations

Abbreviation	Definition
IDA	Iron-deficiency anemia
Hb	Hemoglobin
ID	IFiguewron deficiency
JBI	Joanna Briggs Institute
SWiM	Synthesis Without Meta-analysis
RBC	Red blood cell
WHO	World Health Organization
PRISMA	Preferred Reporting Items for Systematic Reviews and Meta-Analyses
SDL	Saudi Digital Library
